# Antepartum and intrapartum stillbirth rates across gestation: a cross-sectional study using the revised foetal death reporting system in the U.S.

**DOI:** 10.1186/s12884-022-05185-x

**Published:** 2022-11-29

**Authors:** Collette N. Ncube, Sarah M. McCormick, Sylvia E. Badon, Taylor Riley, Vivienne L. Souter

**Affiliations:** 1 Department of Epidemiology, Boston University, School of Public Health, Boston, MA 02118 USA; 2 Department of Obstetrics and Gynecology, Maternal Fetal Medicine, Madigan Army Medical Center, Tacoma, WA USA; 3 Kaiser Permanente Northern California Division of Research, Oakland, CA USA; 4 Department of Epidemiology, School of Public Health, University of Washington, Seattle, WA USA; 5 Department of Health Services, School of Public Health, University of Washington, Seattle, WA USA

**Keywords:** Stillbirth, Foetal death, Cause of death, Intrapartum, Parity, Race factors

## Abstract

**Background:**

There is a renewed call to address preventable foetal deaths in high-income countries, especially where progress has been slow. The Centers for Disease Control and Prevention released publicly, for the first time, the initiating cause and estimated timing of foetal deaths in 2014. The objective of this study is to describe risk and characteristics of antepartum versus intrapartum stillbirths in the U.S., and frequency of pathological examination to determine cause.

**Methods:**

We conducted a cross-sectional study of singleton births (24–43 weeks) using 2014 U.S. Fetal Death and Natality data available from the National Center for Health Statistics. The primary outcome was timing of death (antepartum (*n* = 6200), intrapartum (*n* = 453), and unknown (*n* = 5403)). Risk factors of interest included maternal sociodemographic, behavioural, medical and obstetric factors, along with foetal sex. We estimated gestational week-specific stillbirth hazard, risk factors for intrapartum versus antepartum stillbirth using multivariable log-binomial regression models, conditional probabilities of intrapartum and antepartum stillbirth at each gestational week, and frequency of pathological examination by timing of death.

**Results:**

The gestational age-specific stillbirth hazard was approximately 2 per 10,000 foetus-weeks among preterm gestations and > 3 per 10,000 foetus-weeks among term gestations. Both antepartum and intrapartum stillbirth risk increased in late-term and post-term gestations. The risk of intrapartum versus antepartum stillbirth was higher among those without a prior live birth, relative to those with at least one prior live birth (RR 1.32; 95% CI 1.08–1.61) and those with gestational hypertension, relative to those with no report of gestational hypertension (RR 1.47; 95% CI 1.09–1.96), and lower among Black, relative to white, individuals (RR 0.70; 95% CI 0.55–0.89). Pathological examination was not performed/planned in 25% of known antepartum stillbirths and 29% of known intrapartum stillbirths.

**Conclusion:**

These findings suggest greater stillbirth risk in the late-term and post-term periods. Primiparous mothers had greater risk of intrapartum than antepartum still birth, suggesting the need for intrapartum interventions for primiparous mothers in this phase of pregnancy to prevent some intrapartum foetal deaths. Efforts are needed to improve understanding, prevention and investigation of foetal deaths as well as improve stillbirth data quality and completeness in the United States.

**Supplementary Information:**

The online version contains supplementary material available at 10.1186/s12884-022-05185-x.

## Background

In 2013, over 23,500 stillbirths (intrauterine deaths at ≥20 weeks of gestation, or birthweight ≥350 g if gestational age is unknown [1]) occurred in the U.S. – a stillbirth rate (SBR) of 5.96 foetal deaths per 1000 live births and foetal deaths [2]. Perinatal mortality, defined as stillbirths and neonatal deaths (deaths up to 28 days after birth), has declined in recent years but the SBR has been relatively static, resulting in more foetal deaths than infant deaths (deaths up to 1 year after birth) [2]. Foetal mortality is often overlooked [3] but is a major public health issue, with one out of every 167 U.S. pregnancies that reach 20 weeks’ gestation ending in stillbirth [2]. It is a particularly devastating experience for parents who may experience long-term psychological distress following a stillbirth [3]. Estimated direct financial costs of stillbirth, including medical care, are 10–70% higher than costs incurred for a live birth [4]. Indirect financial costs due to psychological distress, including reduced earnings due to time off work, reduced productivity, or inability to return to paid employment, can be long-lasting [4].

The U.S. is notable for having both a higher SBR and a lower annual decrease in the SBR compared to many other high-income countries [5]. Additionally, racial differences in the SBR in the U.S. are striking with non-Hispanic Black (hereafter referred to as Black) mothers having a SBR more than twice that of non-Hispanic white (hereafter referred to as white) mothers [2]. Despite these unfavourable national statistics, preventing stillbirth has received little public health attention. However, findings from research initiatives such as the Stillbirth Collaborative Research Network are contributing to knowledge on this topic. The public health burden of stillbirth has recently received international attention with calls to address preventable foetal deaths in high-income countries where progress has been slow [5, 6]. The observed variation in SBR across and within high-income countries, the high proportion of foetal deaths classified as unexplained, and the low uptake of interventions believed to be effective in reducing stillbirth risk, are all factors that support the premise that further progress can be made in reducing stillbirth [5]. Opportunities exist in the U.S. to reduce both preventable antepartum *and* intrapartum stillbirths and this should be a priority [7]. Intrapartum stillbirths, defined as foetal deaths occurring during labour and delivery, constitute a minority of stillbirths in high-income countries but represent a target group for stillbirth prevention in countries where foetal monitoring is widely available.

Relatively little information is available on the rate, risk factors for, and causes of intrapartum stillbirths in the U.S., as these losses are rarely distinguished from antepartum stillbirths (foetal deaths occurring prior to labour) [8]. Additionally, there is little research published on differences in maternal and foetal characteristics associated with intrapartum and antepartum stillbirths [8–10]. In 2014, the U.S. National Vital Statistics System (NVSS) Fetal Death File included the following information for the first time: data on the estimated timing of foetal death with respect to labour; whether pathological examination was undertaken; and the cause of foetal death for a subset of states that collected data considered to be of acceptable quality. The goal of this study was to use these data to gain insight into the risk for stillbirth by timing of foetal death (antepartum versus intrapartum), risk factors associated with intrapartum versus antepartum stillbirth, frequency of pathological examination (autopsy and histological placental exam) and the initiating causes of stillbirth among singleton pregnancies.

## Methods

### Study population and data collection

We used 2014 Fetal Death and Live Birth data to conduct a population-based cross-sectional study. We included foetal deaths of U.S. residents in New York City, the District of Columbia and the 41 states (eTable 1) that adopted the Standard Report of Fetal Death 2003 revision by January 1, 2014. This geographic population represents 88% of stillbirths in 2014 [11].

We obtained Fetal Death micro-data files via NVSS [12] and aggregate Live Birth data via the Centers for Disease Control and Prevention (CDC) Wonder natality online databases [13]. Fetal Death data files included information on select maternal sociodemographic, behavioural, medical, and obstetric factors, along with birth characteristics that were potential risk factors for stillbirth. Gestational age at delivery in these data files is based on the obstetric estimate, the best estimate of the infant’s gestation in completed weeks based on the birth attendant’s final estimate of gestation.

The geographic areas mentioned above reported 24,032 stillbirths to U.S. residents, excluding induced terminations of pregnancy [2]. We retained singleton births and excluded observations with gestational ages at delivery < 24 weeks (*n* = 6951) or > 43 weeks (*n* = 4). The former due to variation across states in the reporting requirements of foetal deaths, and likely underreporting at the lower limit of the required reporting period for each state [2]. The latter due to possible implausibility.

We included aggregate data on live births occurring in New York City, the District of Columbia and the 41 states previously mentioned. These areas reported 3,316,293 singleton live births born 24–43 weeks’ gestation.

### Outcome

The outcome variable was stillbirth by timing of foetal death with respect to labour. Estimated timing of death in the NVSS data was categorized as follows: foetus alive at initial assessment and died in labour (intrapartum stillbirth), foetus not alive at initial assessment and not in labour (antepartum stillbirth), foetus not living during labour and no initial assessment was performed (possible intrapartum or antepartum death since the timing of demise with respect to labour onset is unknown), and timing of death not known. Although we included observations from vital statistics jurisdictions that indicated collecting data on estimated timing of foetal death, these data were unknown for some observations. For the purposes of this study, we combined the latter two groups as “unknown timing”. We created a categorical variable for stillbirth: intrapartum stillbirth (*n* = 453), antepartum stillbirth (*n* = 6200), unknown timing (*n* = 5403). Of those where timing of foetal death, with respect to labour, was not known, 19.6% (1061/5403) were in labour at the time when foetal death was first diagnosed and could have been either antepartum or intrapartum stillbirths.

### Exposures

We created categorical variables of the potential risk factors. Maternal sociodemographic factors: maternal age at delivery (< 20, 20–24, 25–34, ≥35 years), educational attainment (<high school diploma, high school diploma/GED, some college of Associate’s degree, ≥Bachelor’s degree, unknown), and race/ethnicity (white, Black, American Indian/Alaska Native, Asian/Pacific Islander, Hispanic). Maternal behavioural factors: timing of first prenatal care visit (first trimester, after first trimester, no care, unknown) and self-reported cigarette smoking at any time during pregnancy (yes, no, unknown). Medical and obstetric factors: parity (primipara, multipara, unknown), pre-pregnancy body mass index (BMI, kg/m^2^), pre-pregnancy diabetes, gestational diabetes, pre-pregnancy hypertension, gestational hypertension, and hypertension eclampsia (yes, no, unknown) [14]. Healthcare provider factors: place of delivery (in-hospital, out of hospital, unknown) and attendant (Doctor of Medicine, Doctor of Osteopathy, Certified Nurse Midwife/Other, unknown). We included foetal sex (male, female) as a foetal characteristic.

We created a combined variable indicating whether pathological examination (either autopsy or histological placental exam) had been performed or planned, versus neither. Initiating causes of death were reported in the death file using International Classification of Diseases tenth revision (ICD-10) classification. The certifier selected one cause of death from the list of conditions and diseases and reported it separately as the initiating cause of death [11]. We created a variable, informed generally by the ICD-PM (Perinatal Mortality) system, grouping the causes by timing of foetal death [15].

### Statistical analysis

In this secondary data analysis, we examined the distribution of potential risk factors among stillbirths. We then fit a multivariable log-binomial regression model with each risk factor (listed in the Exposures section above) as an exposure and timing of stillbirth as the outcome (intrapartum versus antepartum), adjusting for all other listed risk factors as confounders. We estimated risk ratios (RRs) and corresponding 95% confidence intervals (CIs).

Gestational age-specific stillbirth hazard was calculated by timing of foetal death. We approximated the hazard as the number of stillbirths occurring at a specific gestational week, divided by the number of ongoing pregnancies at that gestational week (i.e., live births and stillbirths occurring during that gestational week, plus all foetuses still in utero) [16]. We reported this approximate hazard (incidence density) per 10,000 foetus-weeks.

We calculated the conditional probabilities of intrapartum and antepartum stillbirth for each gestational week. For the conditional probability of intrapartum stillbirth, the numerator was the number of intrapartum stillbirths and the denominator included all births (live births and stillbirths), except antepartum stillbirths, occurring at a specific gestational week. Similarly, for the conditional probability of antepartum stillbirth, the numerator was the number of antepartum stillbirths and the denominator excluded intrapartum stillbirths [17].

We examined the frequency of pathological examination (autopsy and histological placental exam) performed or planned by timing of foetal death overall and separately in stillbirths without congenital malformations. We also examined the initiating causes of stillbirth, by timing of foetal death in a subset of the foetal death data with information on the initiating cause of death (*N* = 9024).

#### Missing data

The NVSS public-use micro-data file provided already imputed maternal race and age. We performed multiple imputation (MI), with 100 imputations, using fully conditional specification to handle the remaining missing data [18] in the main analyses. We assumed the missing data mechanism was ignorable; that is, missing at random (MAR) [19] based upon the observed data. Continuous variables (in the original data files) were modelled using a linear model, and categorical variables were modelled using a logistic model; MI is robust to non-normality [20]. After imputation, we excluded observations originally missing information on timing of foetal death [21]. The multivariable log-binomial regression model was run using each imputed dataset. Results were combined using Rubin’s rules [22].

#### Sensitivity analyses

We conducted a sensitivity analysis imputing for missing outcome values. We performed additional sensitivity analyses including 1) collapsing the category of unknown foetal death timing with antepartum death since the majority of foetal deaths occur antepartum, and 2) excluding foetal deaths affected by congenital anomalies. Obstetric interventions are often withheld in cases of known lethal or serious malformations, potentially affecting foetal monitoring and likelihood of planned pathological examination.

All analyses were conducted using Stata 15.1 (College Station, TX, USA).

## Results

Compared with mothers experiencing an antepartum stillbirth, a higher proportion of mothers experiencing an intrapartum stillbirth had gestational hypertension or no prior live births (primipara); a smaller proportion were Black or had gestational diabetes (Table 1). The overall SBR was 3.62 per 1000 births (live births and foetal deaths) or 1 in 276 births. The rate of known intrapartum stillbirth was 0.14 per 1000 births or 1 in 7142 births. Intrapartum stillbirths constituted 7% of stillbirths with known timing of foetal death and 4% of all stillbirths.

**Table 1:** Foetal and maternal characteristics of stillborn singletons, 24–43 weeks of gestation, in the United States, 2014

	Antepartum deaths ***N*** = 6200		Intrapartum deaths ***N*** = 453		Not living during labour, no initial assessment ^a^ ***N*** = 1061		Unknown timing ***N*** = 4342	
Characteristics	n	%	n	%	n	%	n	%
Male sex	3236	52.2	235	51.9	549	51.7	2753	51.0
Age at delivery (years)								
< 20	434	7.0	40	8.8	89	8.4	344	7.9
20–24	1431	23.1	112	24.7	270	25.5	1045	24.1
25–34	3173	51.2	220	48.6	538	50.7	2162	49.8
≥ 35	1162	18.7	81	17.9	164	15.5	791	18.2
Maternal educational attainment								
< High school diploma	1120	18.1	75	16.6	224	21.1	751	17.3
High school diploma/GED	1819	29.3	140	30.9	332	31.3	1310	30.2
Some college/Associate’s degree	1541	24.9	127	28.0	249	23.5	1019	23.5
≥ Bachelor’s degree	1198	19.3	74	16.3	169	15.9	714	16.4
Unknown	522	8.4	37	8.2	87	8.2	548	12.6
Parity ^b^								
Primipara	2251	36.3	196	43.3	392	37.0	1635	37.7
Multipara	3796	61.2	241	53.2	649	61.2	2496	57.5
Unknown	153	2.5	16	3.5	20	1.9	211	4.9
Timing of first prenatal care visit								
First trimester	4021	64.9	281	62.0	583	55.0	2521	58.1
After first trimester	1303	21.0	99	21.9	244	23.0	860	19.8
No care	325	5.2	25	5.5	116	10.9	304	7.0
Unknown	551	8.9	48	10.6	118	11.1	657	15.1
Maternal smoking ^c^								
Yes	682	11.0	53	11.7	132	12.4	477	11.0
No	4787	77.2	346	76.4	789	74.4	3147	72.5
Unknown	731	11.8	54	11.9	140	13.2	718	16.5
Pre-pregnancy body mass index (kg/m^2^) ^d^								
Underweight	151	2.4	17	3.8	27	2.5	121	2.8
Normal	2028	32.7	143	31.6	385	36.3	1317	30.3
Overweight	1474	23.8	107	23.6	227	21.4	972	22.4
Obese class I	959	15.5	68	15.0	149	14.0	638	14.7
Obese class II	528	8.5	34	7.5	75	7.1	342	7.9
Obese class III	418	6.7	27	6.0	57	5.4	294	6.8
Unknown	642	10.4	57	12.6	141	13.3	658	15.2
Maternal race ^e^								
White	2938	47.4	233	51.4	498	46.9	1923	44.3
Black	1626	26.2	95	21.0	300	28.3	1198	27.6
American Indian/Alaska Native	80	1.3	10	2.2	13	1.2	54	1.2
Asian/Pacific Islander	321	5.2	21	4.6	44	4.2	247	5.7
Hispanic	1235	19.9	94	20.8	206	19.4	920	21.2
Pre-pregnancy diabetes								
Yes	298	4.8	21	4.6	27	2.5	167	3.9
No	5722	92.3	42	92.7	992	93.5	3800	88.7
Unknown	180	2.9	12	2.6	42	3.9	318	7.4
Gestational diabetes								
Yes	404	6.5	21	4.6	43	4.1	209	4.9
No	5616	90.6	420	92.7	976	91.9	3758	87.7
Unknown	180	2.9	12	2.7	42	3.9	318	7.4
Pre-pregnancy hypertension								
Yes	337	5.4	21	4.6	48	4.5	201	4.7
No	5683	91.7	420	92.7	971	91.5	3766	87.9
Unknown	180	2.9	12	2.7	42	3.9	318	7.4
Gestational hypertension								
Yes	453	7.3	48	10.6	72	6.8	275	6.5
No	5567	89.8	393	86.8	947	89.3	3692	86.2
Unknown	180	2.9	12	2.7	42	3.9	318	7.4
Eclampsia								
Yes	49	0.8	5	1.1	5	0.5	34	0.8
No	5971	96.3	436	96.3	1014	95.6	2933	91.8
Unknown	180	2.9	12	2.7	42	3.9	318	7.4
Delivery Place								
In hospital	6109	98.5	442	97.6	1044	98.4	4197	96.7
Out of hospital	83	1.3	10	2.2	17	1.6	141	3.3
Unknown	8	0.2	1	0.2	0	0	4	0.1
Attendant								
Doctor of Medicine	5367	86.6	399	88.1	891	83.9	3713	85.5
Doctor of Osteopathy	464	7.5	28	6.2	92	8.7	299	6.9
Certified Nurse Midwife/Other	264	4.3	17	3.8	62	5.8	238	5.5
Unknown	105	1.7	9	2	16	1.5	92	2.1

Foetal deaths (data source: public use data file) of U.S. residents in New York City, the District of Columbia and the 41 states (see eTable 1)

^a^Foetus not living during labour and no initial assessment was performed (possible intrapartum or antepartum death). ^b^Includes prior live births, now living or dead. ^c^Maternal tobacco use at any time during pregnancy. ^d^Underweight (< 18.5), Normal (18.5–24.9), Overweight (25–29.9), Obese class I (30–34.9), Obese class II (35–39.9), Obese class III (≥40). ^e^ Individuals for whom Hispanic ethnicity was unknown were assumed to be non-Hispanic; white, black, American Indian/Alaska Native and Asian/Pacific Islander are non-Hispanic

Mothers with no prior live birth, relative to those with at least one prior live birth, had greater risk (adjusted RR 1.32; 95% CI 1.08, 1.61) of intrapartum stillbirth than antepartum stillbirth. Black mothers, relative to white mothers, had lower risk (adjusted RR 0.70; 95% CI 0.55, 0.89) of intrapartum stillbirth than antepartum stillbirth. Asian/Pacific Islander and Hispanic mothers also had a lower risk of intrapartum than antepartum stillbirth, while American Indian/Alaska Native had higher risk, relative to white mothers, but the 95% CIs included the null. Mothers with gestational hypertension had a higher risk of intrapartum stillbirth than antepartum stillbirth (adjusted RR 1.47; 95% CI 1.09, 1.96) compared to mothers with no gestational hypertension (Table 2).

**Table 2:** Risk factors for intrapartum stillbirth relative to antepartum stillbirth, 24–43 weeks of gestation, United States 2014

	Intrapartum death	
	Unadjusted RR (95% CI)	Adjusted ^a^ RR (95% CI)
Male sex	0.99 (0.83, 1.18)	0.99 (0.83, 1.18)
Age at delivery (years)		
< 20	1.16 (0.82, 1.64)	1.09 (0.77, 1.54)
20–24	1.00 (Reference)	1.00 (Reference)
25–34	0.89 (0.72, 1.11)	0.99 (0.78, 1.25)
≥ 35	0.90 (0.68, 1.18)	1.06 (0.78, 1.43)
Maternal education < Bachelor’s degree ^b^	1.20 (0.95, 1.53)	1.26 (0.96, 1.66)
Primipara ^c^	1.33 (1.11, 1.59)	1.32 (1.08, 1.61)
Timing of first prenatal care visit		
First trimester	1.00 (Reference)	1.00 (Reference)
After first trimester	1.08 (0.86, 1.35)	1.06 (0.85, 1.33)
No care	1.08 (0.73, 1.60)	1.04 (0.69, 1.54)
Maternal cigarette smoking ^d^	1.06 (0.81, 1.41)	1.02 (0.76, 1.36)
Pre-pregnancy body mass index (kg/m^2^) ^e^		
Underweight	1.46 (0.90, 2.35)	1.43 (0.88, 2.31)
Normal	1.00 (Reference)	1.00 (Reference)
Overweight	1.01 (0.80, 1.29)	1.04 (0.81, 1.32)
Obese class I	0.99 (0.76, 1.32)	1.05 (0.79, 1.39)
Obese class II	0.92 (0.63, 1.32)	0.95 (0.65, 1.38)
Obese class III	0.92 (0.62, 1.36)	0.95 (0.63, 1.44)
Maternal race ^f^		
White	1.00 (Reference)	1.00 (Reference)
Black	0.75 (0.60, 0.95)	0.70 (0.55, 0.89)
American Indian/Alaska Native	1.51 (0.83, 2.75)	1.43 (0.78, 2.60)
Asian/Pacific Islander	0.84 (0.54, 1.29)	0.85 (0.55, 1.31)
Hispanic	0.96 (0.76, 1.21)	0.95 (0.74, 1.21)
Pre-pregnancy diabetes ^g^	0.96 (0.63, 1.47)	0.96 (0.62, 1.48)
Gestational diabetes ^h^	0.71 (0.46, 1.09)	0.71 (0.46, 1.09)
Pre-pregnancy hypertension ^i^	0.85 (0.56, 1.30)	0.96 (0.62,1.50)
Gestational hypertension ^j^	1.45 (1.09, 1.93)	1.47 (1.09, 1.96)
Eclampsia ^k^	1.34 (0.58, 3.12)	1.25 (0.54, 2.91)
In-hospital delivery ^l^	0.63 (0.35, 1.14)	0.64 (0.35,1.17)
Attendant		
Doctor of Medicine	1.00 (Reference)	1.00 (Reference)
Doctor of Osteopathy	0.82 (0.57, 1.19)	0.79 (0.54, 1.15)
Certified Nurse Midwife/Other	0.87 (0.54, 1.40)	0.78 (0.48, 1.26)

*RR* risk ratio

^a^Adjusted for all other risk factors in the table. Antepartum stillbirths are the comparison group. ^b^Reference group is ≥ Bachelor’s degree. ^c^Parity includes prior live births, now living or dead; reference group is multipara. ^d^Maternal cigarette use at any time during pregnancy. Reference category is no smoking. ^e^Underweight (< 18.5), Normal (18.5–24.9), Overweight (25–29.9), Obese class I (30–34.9), Obese class II (35–39.9), Obese class III (≥40). ^f^Individuals for whom Hispanic ethnicity was unknown were assumed to be non-Hispanic. ^g-k^Reference group includes those without a reported diagnosis of the condition ^l^Reference group is out of hospital place of delivery

**Fig. 1:**
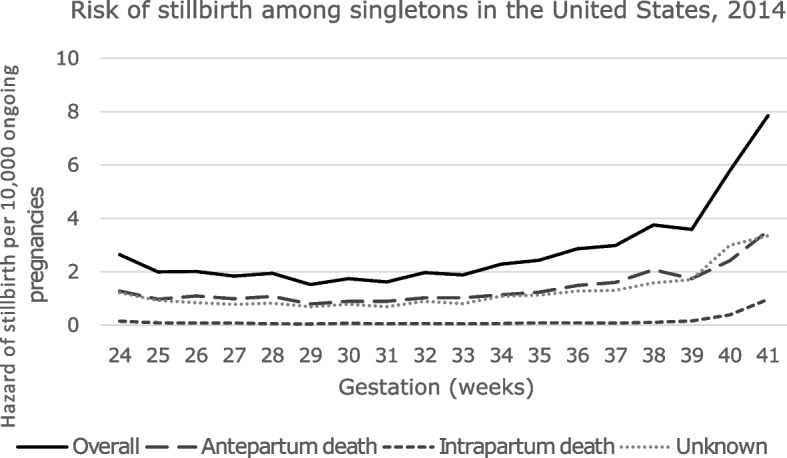
Gestational age-specific risk of stillbirth (per 10,000 foetus-weeks) among singletons in the United States by timing of foetal death (antepartum, intrapartum, unknown), 2014

The gestational age-specific stillbirth hazard increased from a nadir of 1.52 per 10,000 foetus-weeks at 29 weeks’ gestation, rising gradually to 3.59 per 10,000 at 39 weeks, and 7.85 per 10,000 foetus-weeks at 41 weeks (Fig. 1). This rise in overall stillbirth risk was due to an increase in antepartum *and* intrapartum stillbirth risk. The gestational age-specific antepartum and intrapartum stillbirth hazards increased from a nadir of 0.79 and 0.05 per 10,000 ongoing pregnancies at 29 weeks’ gestation to 3.53 and 0.96 per 10,000 pregnancies, respectively, at 41 weeks (Fig. 1).

**Fig. 2:**
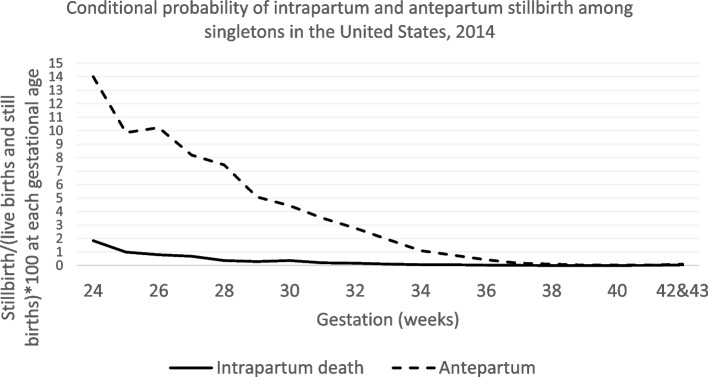
Conditional probabilities of intrapartum and antepartum stillbirth among individuals giving birth to singletons at each gestational age, 2014

The conditional probability of intrapartum stillbirth decreased from 1.8% at 24 weeks to a low of 0.003% at 39 weeks, while the conditional probability of antepartum stillbirth decreased from a high of 14% at 24 weeks to 0.03% at 39 weeks. At 40 weeks, the conditional probability of intrapartum stillbirth increased again and was 0.04% (1 in 2273 births) at 42–43 weeks (Fig. 2, eTable 2). The conditional probability of antepartum stillbirth increased only slightly in the post-term period.

Histology of the placenta was performed/planned for the majority of stillbirths; whereas an autopsy was performed/planned for a minority. About a quarter of antepartum stillbirths, 28.7% of intrapartum stillbirths and 33.6% of those with unknown timing of foetal death had neither autopsy nor pathological evaluation of the placenta. The proportion of stillbirths with performed/planned histological examination or autopsy was lower among foetuses diagnosed with congenital malformations (Table 3). An initiating cause of death was recorded for 4850 antepartum (78.2%) and 364 intrapartum (80.4%) stillbirths. Of these, about 9% of antepartum stillbirths and 27.8% of intrapartum stillbirths were associated with foetal abnormalities. The initiating cause was “unspecified” in 33.1% of antepartum and 11.8% of intrapartum stillbirths (Table 4).

**Table 3:** Pathological examination among singleton stillbirths 24–43 weeks of gestation, by timing of foetal death, United States 2014

	Antepartum deaths ***N*** = 6200		Antepartum deaths without CM ^b^ ***N*** = 558		Intrapartum deaths ***N*** = 453		Intrapartum deaths without CM ^b^ ***N*** = 122		Unknown timing ^c^ ***N*** = 5403	
	n	%	n	%	n	%	n	%	n	%
Autopsy										
Performed	912	14.7	74	13.3	63	13.9	13	10.7	850	15.7
Planned	642	10.4	44	7.9	28	6.2	6	4.9	479	8.9
Neither	4646	74.9	440	78.9	362	79.9	103	84.4	4017	74.4
Unknown									57	1.1
Histological placental exam										
Performed	3145	50.7	255	45.7	240	53.0	50	41.0	2412	44.6
Planned	1320	21.3	112	20.1	73	16.1	22	18.0	956	17.7
Neither	1735	28.0	191	34.2	140	30.9	50	41.0	1978	36.6
Unknown									57	1.1
Neither autopsy nor histological placental exam ^a^	1562	25.2	176	31.5	130	28.7	49	40.2	1813	33.6

^a^ Neither autopsy nor histological placental examination were performed or the status of both examinations is unknown. ^b^ Congenital malformations, deformations and chromosomal abnormalities. ^C^ Includes those not living during labour and no initial assessment was performed, and those of unknown timing of death

**Table 4:** Initiating causes of stillbirth in the United States, by timing of foetal death, 2014

Antepartum death (*N* = 4850)	**n**	**%**
A1: Congenital malformations, deformations and chromosomal abnormalities	458	9.4
A2: Infection	3	0.1
A3: Antepartum hypoxia	11	0.2
A4: Other specified antepartum disorder	271	5.6
A5: Disorders related to foetal growth	33	0.7
A6: Antepartum death of unspecified cause	1604	33.1
M1: Complications of placenta, cord and membranes	1701	35.1
M2: Maternal complications of pregnancy	173	3.6
M3: Other complications of labour and delivery	39	0.8
M4: Maternal medical and surgical conditions	444	9.2
Unknown	113	2.3
Intrapartum death (*N* = 364)	**n**	**%**
I1: Congenital malformations, deformations and chromosomal abnormalities	101	27.8
I2: Birth trauma	0	
I3: Acute intrapartum event	1	0.3
I4: Infection	1	0.3
I5: Other specified intrapartum disorder	13	3.6
I6: Disorders related to foetal growth	7	1.9
I7: Intrapartum death of unspecified cause	43	11.8
M1: Complications of placenta, cord and membranes	111	30.5
M2: Maternal complications of pregnancy	35	9.6
M3: Other complications of labour and delivery	14	3.9
M4: Maternal medical and surgical conditions	32	8.8
Unknown	6	1.7

Of the 12,056 foetal deaths in this study, 9024 were in areas that collected data on initiating cause of foetal death. There were 3810 stillbirths where the timing of foetal death in relation to labour was unknown, a combination of those not living during labour and no initial assessment was performed, and those with unknown timing of death

## Discussion

### Principal findings

Using national data to understand the risk factors for stillbirth in the U.S. is crucial in the effort to prevent stillbirth. A lack of national data on *intrapartum* stillbirth was highlighted as an area of concern in the effort to reduce SBRs [23]. The release of publicly available data distinguishing intrapartum and antepartum stillbirths in the 2014 Fetal Death file has enabled us to explore intrapartum and antepartum stillbirth risk across gestation, differences in risk factors for intrapartum stillbirth relative to antepartum stillbirth, whether pathological examination was undertaken, and the initiating cause of stillbirth. Our study has shown that the risk for intrapartum stillbirth increases in the late-term period, and mothers without a prior live birth (compared to mothers with at least one prior live birth) and mothers with gestational hypertension (compared to mothers without reported gestational hypertension) are at increased risk for intrapartum compared to antepartum stillbirth, even after adjusting for confounding factors. We showed that Black mothers, who are over-represented in stillbirths in general, appear to be less at risk of intrapartum compared to antepartum stillbirth, relative to white mothers. However, exclusion of stillbirths less than 24 weeks of gestation, when the Black-white disparity is greatest, may explain this finding [24]. A minority of stillbirths are investigated via autopsy or placental pathological examination, and foetal death record data regarding the initiating cause(s) of stillbirth are limited.

### Strengths of the study

We used a database with substantial geographic and ethnic diversity, capturing the majority of stillbirths in the US, and we had the ability to differentiate between antepartum and intrapartum timing of stillbirth. We restricted the gestational age range from 24 to 43 weeks and required data on estimated timing of foetal death, which may explain the lower overall SBR in this study than that reported by NVSS for 2014 [11]. While being able to distinguish intrapartum from antepartum stillbirths may not be unique in studies from other countries [17], the current study using US national foetal death data is unique due to the only recent public availability of these data.

### Limitations of the data

Because gestational age in the Fetal Death file was available in completed weeks, rather than days, we approximated the stillbirth hazard by calculating an incidence density at each gestational week among those at risk for stillbirth at the beginning of that gestational week onward. We excluded foetal deaths < 24 weeks of gestation due to low viability and therefore little opportunity for prevention. We were also concerned about variation across states in reporting requirements and likely underreporting at the lower limit of the required reporting period for each state. While the majority of states require reporting at ≥20 weeks gestation or 350 g (which is approximately 20 weeks), a handful of states require reporting at slightly later gestations [25]. Since underreporting "is most likely to occur in the earlier part of the required reporting period," [11] we chose a cut-off of 24 weeks. Additionally, we restricted the study to singleton births. Multiple pregnancies represent only 2–3% of births in the US and are different from singleton pregnancies in their risk for stillbirth, gestational age at delivery, and potential for pregnancy complications (such as twin-to-twin transfusion syndrome). Due to this we believe SBRs should be reported separately for singletons and multiple pregnancies. As a result, total SBRs cannot be directly compared with other U.S. studies and those reporting stillbirths from 20 weeks gestation.

Risk factors for antepartum and intrapartum foetal death, relative to live births could not be explored due to differences in the geographic areas included in the Fetal Death micro-data files and those in the birth micro-data files. The geographic locations that adopted the Standard Report of Live Birth 2003 revision by the beginning of 2014 (i.e., individual records in the 2014 Live Birth public use file) were not the same geographic locations that had also adopted the Standard Report of Fetal Death 2003 revision (i.e., individual records in the 2014 Fetal Death public use file). Thus, we were only able to explore differences in risk factor prevalence for intrapartum compared to antepartum stillbirth. Future research should explore risk factors for antepartum and intrapartum foetal deaths, as compared with live births, to better inform clinical practice.

The data on cause of foetal death in our study are limited for a number of reasons. First, the ICD-10 is restricted in the diagnostic categories included for stillbirth [26]. In addition, post-mortem evaluation, one of the key investigations in establishing the cause of foetal death, was missing in many cases [27], and even if post-mortem was performed, the results may not have been available at the time of completion of the death certificate. The investigation of stillbirth included only pathological examination as information on maternal evaluation and genetic testing/DNA banking is not available in the foetal death record. Consequently, the cause of recorded death may be incorrect in as many as half of cases when compared to the clinical records [28].

We observed a large proportion of stillbirths for whom the timing of death was unknown, additionally, up to 15% of the sample was missing data on risk factors examined (Table 1). For the main analyses, we multiply imputed missing data under the assumption of MAR; however, it is possible that the data were missing not at random (MNAR). We examined the potential mechanisms associated with missingness and found that missingness of the outcomes was correlated with missingness of other covariates (eTable 3). Vital statistics data are known to suffer from incomplete or misreported data [2, 29]; however, we are not aware of studies at the state or national level that report on the quality of foetal death data with respect to information on the time of death. Given the emotional distress associated with foetal death, obtaining self-reported data is likely challenging and less actively pursued when compared to obtaining the comparable information for live births. MI in the presence of data MNAR may produce biased results, the magnitude or direction of which cannot be estimated from the data [30]. The results of the sensitivity analyses did not differ substantially from the main findings as to affect inferences made (eTable 3).

The year 2014 was the first time publicly-available Fetal Death files included information on the estimated timing of foetal death with respect to labour, whether pathological examination was undertaken, and the cause of foetal death. This served as the impetus to use these data. Since that inaugural year, time has lapsed and the most recently available data (as of the completion of this work) are for the year 2020. By 2020, all US states and territories had adopted the Standard Report of Fetal Death 2003 revision; this is in comparison with New York City, the District of Columbia and the 41 states (eTable 1) that had adopted the new reporting system as of 2014. This development would result in the retention of more records for the analytic sample from jurisdictions reporting estimated timing of foetal death. The anticipated effect of this would be an SBR less discrepant from the national SBR. With little change in the national SBR in recent years (596.0 in 2013, 597.5 in 2014, and 594.8 foetal deaths per 1000 live births and foetal deaths in 2015–17) [2, 11, 31], and no evidence at present of relevant changes to obstetric practice or major shifts in maternal sociodemographic factors between 2014 and 2020, we would not anticipate substantially different results in follow up studies using more recent data. That being said, future work examining trends in risk of antepartum and intrapartum stillbirth, and the explanatory role of various risk factors, would be a valuable contribution to the field. Because the jurisdictions that were late adopters of the Standard Report of Fetal Death 2003 revision may differ in important ways, future studies should take this into account when examining changes over time.

### Interpretation

The finding that risk for intrapartum stillbirth increases in the late-term period is important because while the increased risk of stillbirth after 39 weeks is well recognized [32], the contribution of intrapartum stillbirth to these losses is not. A recent perinatal audit from the Netherlands also reported an increased risk for intrapartum stillbirth in pregnancies of ≥ 41 weeks and highlighted the contribution of intrapartum hypoxia and substandard care in these losses [33]. When employing antepartum surveillance in high-risk or advanced maternal age populations, lower rates of stillbirth have been observed [34, 35], and current evidence also suggests that there are lower rates of *intrapartum* foetal death when foetal monitoring is utilized in certain populations [36].

Although we found that Black mothers have lower risk of intrapartum compared to antepartum stillbirth relative to white mothers, Black mothers are over-represented among those experiencing both antepartum and intrapartum stillbirths. Antepartum and intrapartum interventions are necessary to reduce Black-white disparities in stillbirth risk.

Not unlike a UK study [37], in our study we found data from foetal death records regarding the initiating cause of stillbirth to be limited. Our study highlights ongoing deficiencies in the investigation of stillbirth in the U.S., with autopsy being planned/performed in only a minority of cases and almost 1 in 3 stillbirths having no placental pathology examination. Similar to our results, a records review of 5 years of foetal death certificates in Utah revealed that autopsy was performed in only 25% of cases and placental evaluation in approximately half of cases [28]. Autopsy and placental pathology examination can provide information about the cause of stillbirth, recurrence risk and possible preventability in future pregnancies [27] but uptake of this testing was far from universal in our study population. Flenady et al. found that only 1/3 of providers in high-income countries reported autopsy was performed for stillbirth [5]. It is not known whether autopsy and placental evaluation were offered and subsequently declined by the patient. Decline of autopsy or placental examination may represent patient cultural beliefs or personal choice, a failure of clinicians to offer autopsy or discuss its value [38], lack of access to perinatology services, or direct cost to the patient [39, 40]. When evaluating usefulness of diagnostic tests, placental pathologic examination and foetal autopsy identify the cause of foetal death in 65 and 42% of cases, respectively; these far outweigh other testing options that included genetic testing, antiphospholipid antibodies, feto-maternal haemorrhage, and glucose screening [27]. The American College of Obstetricians and Gynecologists recommendations support gross and microscopic examination of the placenta, umbilical cord and membranes as an essential component of the evaluation of any stillbirth [36].

## Conclusions

The SBR in the U.S. is high relative to comparable high-income countries [5] and is notable for its wide variation by race [2]. Information about the risk factors for and the causes of stillbirth is crucial to developing strategies to reduce the SBR. Intrapartum stillbirths, in which the foetus is alive at the time of first assessment and then subsequently dies during the birth process, represent a minority of stillbirths in industrialized countries but may reflect the quality of intrapartum care and have high potential for preventability [41]. We hope that the results of our study, and the greater use and scrutiny of these data, will in time drive improvements in the standardization of stillbirth reporting, data quality, and the content of foetal death certificate reports. Our results highlight the presence of opportunities to improve care (including greater use of autopsy and placental pathology) and reduce stillbirth rates and racial disparities in the U.S.

## Supplementary Information

**Additional file 1.**

## Data Availability

The datasets analysed for this study are publicly available from the National Center for Health Statistics (https://www.cdc.gov/nchs/data_access/vitalstatsonline.htm#Fetal_Death) and the Centers for Disease Control and Prevention (https://wonder.cdc.gov/Natality.html). Statistical code used in our analyses are available from https://github.com/c-ncube/ante_intrapartum_stillbirth.

## Abbreviations

SBR: Stillbirth rate
NVSS: National Vital Statistics System
CDC: Centers for Disease Control and Prevention
BMI: Body mass index
ICD: International Classification of Diseases
RR: Rate ratios
CI: Confidence intervals
MI: Multiple imputation
MAR: Missing at random
MNAR: Missing not at random

## References

[1] American College of Obstetricians and Gynecologists, Society for Maternal-Fetal Medicine. Management of Stillbirth: obstetric care consensus no, 10. Obstet Gynecol 2020;135(3):e110-e132. 10.1097/AOG.0000000000003719. 10.1097/AOG.0000000000003719 32080052

[2] Macdorman MF, Gregory ECW. Fetal and perinatal mortality: United States, 2013. Natl Vital Stat Rep. 2013;64(8) Accessed 24Aug 2021. https://www.cdc.gov/nchs/data/nvsr/nvsr64/nvsr64_08.pdf. 26222771

[3] Frøen JF, Cacciatore J, McClure EM (2011). Stillbirths: why they matter. Lancet..

[4] Heazell AEP, Siassakos D, Blencowe H (2016). Stillbirths: economic and psychosocial consequences. Lancet..

[5] Flenady V, Wojcieszek AM, Middleton P (2016). Stillbirths: recall to action in high-income countries. Lancet..

[6] Flenady V, Middleton P, Smith GC (2011). Stillbirths: the way forward in high-income countries. Lancet..

[7] Flenady V, Koopmans L, Middleton P (2011). Major risk factors for stillbirth in high-income countries: a systematic review and meta-analysis. Lancet..

[8] Bodnar LM, Parks WT, Perkins K (2015). Maternal prepregnancy obesity and cause-specific stillbirth. Am J Clin Nutr.

[9] Ananth CV, Savitz DA, Bowes WA (1995). Hypertensive disorders of pregnancy and stillbirth in North Carolina, 1988 to 1991. Acta Obstet Gynecol Scand.

[10] Getahun D, Ananth CV, Kinzler WL (2007). Risk factors for antepartum and intrapartum stillbirth: a population-based study. Am J Obstet Gynecol.

[11] Hoyert DL, Gregory ECW. Cause of fetal death: data from the fetal death report, 2014. Natl Vital Stat Rep 2016;65(7). Accessed 24 Aug 2021. https://www.cdc.gov/nchs/data/nvsr/nvsr65/nvsr65_07.pdf. 27805550

[12] National Center for Health Statistics. 2014 Fetal death data set: public-use data file and documentation. Published 2016. . https://www.cdc.gov/nchs/data_access/vitalstatsonline.htm#Fetal_Death.

[13] Centers for Disease Control and Precention. CDC Wonder Natality Data. Accessed June 29, 2017. https://wonder.cdc.gov/Natality.html.

[14] Simpson LL (2002). Maternal medical disease: risk of antepartum fetal death. Semin Perinatol.

[15] Allanson ER, Tunçalp Ö, Gardosi J (2016). The WHO application of ICD-10 to deaths during the perinatal period (ICD-PM): results from pilot database testing in South Africa and United Kingdom. BJOG..

[16] Kramer MS, Liu S, Luo Z, Yuan H, Platt RW, Joseph KS (2002). Analysis of perinatal mortality and its components: time for a change?. Am J Epidemiol.

[17] Smith GS (2001). Life-table analysis of the risk of perinatal death at term and post term in singleton pregnancies. Am J Obstet Gynecol.

[18] van Buuren S, Brand JPL, Groothuis-Oudshoorn CGM, Rubin DB (2006). Fully conditional specification in multivariate imputation. J Stat Comput Simul.

[19] Rubin DB (1976). Inference and missing data. Biometrika..

[20] Lee KJ, Carlin JB (2010). Multiple imputation for missing data: fully conditional specification versus multivariate normal imputation. Am J Epidemiol.

[21] von Hippel PT (2007). 4. Regression with missing Ys: an improved strategy for analyzing multiply imputed data. Sociol Methodol.

[22] Rubin DB (1987). Multiple imputation for nonresponse in surveys.

[23] Lawn JE, Blencowe H, Waiswa P (2016). Stillbirths: rates, risk factors, and acceleration towards 2030. Lancet..

[24] Willinger M, Ko CW, Reddy UM (2009). Racial disparities in stillbirth risk across gestation in the United States. Am J Obstet Gynecol.

[25] National Center for Health Statistics. User Guide to the 2014 Fetal Death Public Use File; 2016. Accessed 28 June 2022. https://www.cdc.gov/nchs/data_access/vitalstatsonline.htm.

[26] World Health Organization. The WHO application of ICD-10 to deaths during the perinatal Period*:* ICD-PM.; 2016. Accessed 30 Aug 2021. https://www.who.int/reproductivehealth/publications/monitoring/icd-10-perinatal-deaths/en/.

[27] Page JM, Christiansen-Lindquist L, Thorsten V (2017). Diagnostic tests for evaluation of stillbirth: results from the stillbirth collaborative research network. Obstet Gynecol.

[28] Heuser CC, Hunn J, Varner M, Hossain S, Vered S, Silver RM (2010). Correlation between stillbirth vital statistics and medical records. Obstet Gynecol.

[29] Christiansen-Lindquist L, Silver RM, Parker CB (2017). Fetal death certificate data quality: a tale of two U.S. counties. Ann Epidemiol.

[30] Sterne JAC, White IR, Carlin JB (2009). Multiple imputation for missing data in epidemiological and clinical research: potential and pitfalls. BMJ..

[31] Hoyert DL, Gregory ECW. Cause-of-death Data From the Fetal Death File, 2015–2017. 2020;69(4). https://www.cdc.gov/nchs/products/index.htm. 32510316

[32] Rosenstein MG, Cheng YW, Snowden JM, Nicholson JM, Caughey AB (2012). Risk of stillbirth and infant death stratified by gestational age. Obstet Gynecol.

[33] Kortekaas JC, Scheuer AC, de Miranda E, et al. Perinatal death beyond 41 weeks pregnancy: an evaluation of causes and substandard care factors as identified in perinatal audit in the Netherlands. BMC Pregnancy Childbirth. 2018;18(1):1–9. 10.1186/S12884-018-1973-0. 10.1186/s12884-018-1973-0 PMC6149052 30236080

[34] Clark SL, Sabey P, Jolley K (1989). Nonstress testing with acoustic stimulation and amniotic fluid volume assessment: 5973 tests without unexpected fetal death. Am J Obstet Gynecol.

[35] Fox NS, Rebarber A, Silverstein M, Roman AS, Klauser CK, Saltzman DH (2013). The effectiveness of antepartum surveillance in reducing the risk of stillbirth in patients with advanced maternal age. Eur J Obstetr Gynecol Reprod Biol.

[36] American College of Obstetricians and Gynecologists’ Committee on Practice Bulletins—Obstetrics. Antepartum fetal surveillance: ACOG practice bulletin summary, number 229. Obstet Gynecol 2021;137(6):1134–1136. 10.1097/AOG.0000000000004411. 10.1097/AOG.0000000000004411 34011881

[37] Cockerill R, Whitworth MK, Heazell AEP (2012). Do medical certificates of stillbirth provide accurate and useful information regarding the cause of death?. Paediatr Perinat Epidemiol.

[38] Miller ES, Minturn L, Linn R, Weese-Mayer DE, Ernst LM (2016). Stillbirth evaluation: a stepwise assessment of placental pathology and autopsy. Am J Obstet Gynecol.

[39] Michalski ST, Porter J, Pauli RM (2002). Costs and consequences of comprehensive stillbirth assessment. Am J Obstet Gynecol.

[40] Mistry H, Heazell AEP, Vincent O, Roberts T. A structured review and exploration of the healthcare costs associated with stillbirth and a subsequent pregnancy in England and Wales. BMC Pregnancy Childbirth. 2013;13(1):1–11. 10.1186/1471-2393-13-236. 10.1186/1471-2393-13-236 PMC3878511 24341329

[41] Draper E, Kurinczuk JJ, Kenyon S, on behalf of MBRRACE-UK, eds. MBRRACE-UK 2017 Perinatal Confidential Enquiry: Term, Singleton, Intrapartum Stillbirth and Intrapartum-Related Neonatal Death. The Infant Mortality and Morbidity Studies, Department of Health Sciences, University of Leicester; 2017.

