# Supplementary material for: Antepartum and intrapartum stillbirth rates across gestation: a cross-sectional study using the revised foetal death reporting system in the U.S
Source: BMC Pregnancy Childbirth. 2022 Nov 29;22:885. doi: 10.1186/s12884-022-05185-x (PMC9706921; doi:10.1186/s12884-022-05185-x)
Supplement: Supplementary file 1 — Additional file 1. [file 12884_2022_5185_MOESM1_ESM.docx]

**eTable 1:** States that adopted the Standard Report of Fetal Death 2003 revision by January 1, 2014

Alabama, Arizona, Arkansas, California, Delaware, Florida, Georgia, Hawaii, Idaho, Illinois, Indiana, Iowa, Kansas, Kentucky, Louisiana, Maine, Maryland, Michigan, Minnesota, Mississippi, Missouri, Montana, Nebraska, Nevada, New Hampshire, New Mexico, North Carolina, North Dakota, Ohio, Oklahoma, Oregon, Pennsylvania, South Carolina, South Dakota, Tennessee, Texas, Utah, Vermont, Washington, Wisconsin, and Wyoming.

**eTable 2:** Proportion of intrapartum and antepartum stillbirths among individuals giving birth at each gestational age, 2014

| **Gestational age at delivery** | **Intrapartum stillbirths** | **Antepartum stillbirths** | **Live births** | **Intrapartum**  **Proportion, per 100 ^a^** | **Antepartum Proportion, per 100 ^b^** |
| --- | --- | --- | --- | --- | --- |
| 24 weeks | 48 | 417 | 2560 | 1.840 | 14.007 |
| 25 weeks | 29 | 317 | 2899 | 0.990 | 9.857 |
| 26 weeks | 25 | 356 | 3124 | 0.794 | 10.230 |
| 27 weeks | 25 | 323 | 3616 | 0.687 | 8.200 |
| 28 weeks | 16 | 347 | 4304 | 0.370 | 7.461 |
| 29 weeks | 14 | 256 | 4775 | 0.292 | 5.088 |
| 30 weeks | 23 | 288 | 6197 | 0.370 | 4.441 |
| 31 weeks | 16 | 286 | 7847 | 0.203 | 3.517 |
| 32 weeks | 20 | 329 | 11546 | 0.173 | 2.771 |
| 33 weeks | 17 | 329 | 16721 | 0.102 | 1.930 |
| 34 weeks | 20 | 363 | 32517 | 0.061 | 1.104 |
| 35 weeks | 26 | 388 | 50650 | 0.051 | 0.760 |
| 36 weeks | 27 | 464 | 107705 | 0.025 | 0.429 |
| 37 weeks | 24 | 479 | 275489 | 0.009 | 0.174 |
| 38 weeks | 27 | 563 | 541774 | 0.005 | 0.104 |
| 39 weeks | 33 | 380 | 1241109 | 0.003 | 0.031 |
| 40 weeks | 36 | 226 | 720688 | 0.005 | 0.031 |
| 41 weeks | 21 | 77 | 204173 | 0.010 | 0.038 |
| 42& 43 weeks | 6 | 12 | 13586 | 0.044 | 0.088 |
| ^a^ Intrapartum still births divided by live births plus intrapartum stillbirths delivered at that gestational age | | | | | |

**eTable 3**: Risk factors for intrapartum stillbirth relative to antepartum stillbirth, United States, 2014 – sensitivity analyses

|  | **Intrapartum death** | | | | | |
| --- | --- | --- | --- | --- | --- | --- |
|  | **Sensitivity Analysis 1: Impute missing values for outcome** | | **Sensitivity Analysis 2: Collapse unknown foetal death timing with antepartum death** | | **Sensitivity Analysis 3: Exclude congenital malformations** | |
|  | **Unadjusted**  **RR (95% CI)** | **Adjusted** ^a^  **RR (95% CI)** | **Unadjusted**  **RR (95% CI)** | **Adjusted** ^a^  **RR (95% CI)** | **Unadjusted**  **RR (95% CI)** | **Adjusted** ^a^  **RR (95% CI)** |
| Male sex | 0.99 (0.83. 1.17) | 0.98 (0.82, 1.17) | 1.01 (0.84, 1.21) | 1.01 (0.84, 1.21) | 0.98 (0.79, 1.20) | 0.96 (0.78, 1.19) |
| Age at delivery (years) |  |  |  |  |  |  |
| < 20 | 1.12 (0.81, 1.54) | 1.04 (0.75, 1.43) | 1.13 (0.79, 1.60) | 1.08 (0.76, 1.55) | 1.08 (0.72, 1.62) | 1.03 (0.68, 1.55) |
| 20-24 | 1.00 (Reference) | 1.00 (Reference) | 1.00 (Reference) | 1.00 (Reference) | 1.00 (Reference) | 1.00 (Reference) |
| 25-34 | 0.90 (0.74, 1.11) | 1.01 (0.81, 1.25) | 0.92 (0.74, 1.15) | 1.01 (0.79, 1.28) | 0.82 (0.64, 1.06) | 0.96 (0.78, 1.26) |
| ≥ 35 | 0.87 (0.67, 1.13) | 1.04 (0.78, 1.39) | 0.94 (0.71, 1.25) | 1.08 (0.79, 1.47) | 0.87 (0.63, 1.20) | 1.09 (0.77, 1.56) |
| Educational attainment |  |  |  |  |  |  |
| < Bachelor’s degree | 1.22 (0.94, 1.57) | 1.25 (0.94, 1.66) | 1.13 (0.89, 1.45) | 1.22 (0.92, 1.61) | 1.44 (1.06, 1.95) | 1.52 (1.08, 2.13) |
| ≥ Bachelor’s degree | 1.00 (Reference) | 1.00 (Reference) | 1.00 (Reference) | 1.00 (Reference) | 1.00 (Reference) | 1.00 (Reference) |
| Parity ^b^ |  |  |  |  |  |  |
| Primipara | 1.34 (1.11, 1.60) | 1.34 (1.09, 1.63) | 1.29 (1.08, 1.56) | 1.42 (1.15, 1.76) | 1.42 (1.15, 1.76) | 1.42 (1.13, 1.79) |
| Multipara | 1.00 (Reference) | 1.00 (Reference) | 1.00 (Reference) | 1.00 (Reference) | 1.00 (Reference) | 1.00 (Reference) |
| Timing of first prenatal care visit | |  |  |  |  |  |
| First trimester | 1.00 (Reference) | 1.00 (Reference) | 1.00 (Reference) | 1.00 (Reference) | 1.00 (Reference) | 1.00 (Reference) |
| After first trimester | 1.09 (0.88, 1.37) | 1.08 (0.86, 1.36) | 1.04 (0.83, 1.30) | 1.12 (0.87, 1.45) | 1.13 (0.87, 1.46) | 1.09 (0.84, 1.42) |
| No care | 1.13 (0.75, 1.69) | 1.09 (0.72, 1.64) | 0.86 (0.57, 1.28) | 1.25(0.82, 1.91) | 1.24 (0.80, 1.92) | 1.14 (0.74, 1.76) |
| Maternal smoking ^c^ |  |  |  |  |  |  |
| Yes | 1.09 (0.82, 1.45) | 1.04 (0.77, 1.39) | 1.03 (0.78, 1.36) | 1.17 (0.86, 1.60) | 1.17 (0.86, 1.60) | 1.04 (0.75, 1.45) |
| No | 1.00 (Reference) | 1.00 (Reference) | 1.00 (Reference) | 1.00 (Reference) | 1.00 (Reference) | 1.00 (Reference) |
| Pre-pregnancy body mass index (kg/m^2^) ^d^ | |  |  |  |  |  |
| Underweight | 1.48 (0.94, 2.34) | 1.44 (0.91, 2.27) | 1.36 (0.84, 2.22) | 1.36 (0.83, 2.21) | 1.42 (0.81, 2.50) | 1.37 (078, 2.43) |
| Normal | 1.00 (Reference) | 1.00 (Reference) | 1.00 (Reference) | 1.00 (Reference) | 1.00 (Reference) | 1.00 (Reference) |
| Overweight | 1.04 (0.81, 1.34) | 1.06 (0.82, 1.36) | 1.02 (0.80, 1.33) | 1.04 (0.81, 1.33) | 1.04 (0.78, 1.39) | 1.07 (0.79, 1.43) |
| Obese class I | 0.99 (0.75, 1.33) | 1.03 (0.77, 1.37) | 1.00 (0.75, 1.32) | 1.02 (0.77, 1.36) | 0.98 (0.70, 1.37) | 1.01 (0.72, 1.42) |
| Obese class II | 0.95 (0.68, 1.32) | 0.95 (0.64, 1.41) | 0.94 (0.65, 1.35) | 0.95 (0.65, 1.38) | 1.05 (0.70, 1.57) | 1.04 (0.69, 1.58) |
| Obese class III | 0.93 (0.60, 1.42) | 0.94 (0.64, 1.41) | 0.91 (0.61, 1.35) | 0.90 (0.60, 1.37) | 1.12 (0.72, 1.72) | 1.10 (0.70, 1.73) |
| Maternal race ^e^ |  |  |  |  |  |  |
| White | 1.00 (Reference) | 1.00 (Reference) | 1.00 (Reference) | 1.00 (Reference) | 1.00 (Reference) | 1.00 (Reference) |
| Black | 0.87 (0.71, 1.07) | 0.83 (0.67, 1.03) | 0.71 (0.56, 0.89) | 0.68 (0.53, 0.86) | 0.80 (0.62, 1.03) | 0.72 (0.55, 0.94) |
| American Indian/Alaska Native | 1.32 (0.74, 2.23) | 1.27 (0.71, 2.27) | 1.53 (0.83, 2.8) | 1.52 (0.82, 2.81) | 1.83 (0.97, 3.44) | 1.66 (0.88, 3.130) |
| Asian/Pacific Islander | 0.88 (0.59, 1.30) | 0.89 (0.60, 1.32) | 0.80 (0.51, 1.24) | 0.79 (0.50, 1.23) | 0.79 (0.47, 1.32) | 0.82 (0.49, 1.39) |
| Hispanic | 0.96 (0.76, 1.21) | 0.95 (0.74, 1.22) | 0.92 (0.73, 1.16) | 0.89 (0.69, 1.13) | 0.77 (0.57, 1.03) | 0.74 (0.54, 1.01) |
| Pre-pregnancy diabetes | 0.96 (0.63, 1.48) | 0.97 (0.62, 1.50) | 1.076(0.69, 1.63) | 1.07 (0.69, 167) | 1.08 (0.67, 1.73) | 1.02 (0.63, 1.66) |
| Gestational diabetes | 0.70 (0.46, 1.06) | 0.71 (0.47, 1.08) | 0.79 (0.52, 1.23) | 0.79 (0.51, 1.23) | 0.77 (0.48, 1.24) | 0.76 (0.47, 1.25) |
| Pre-pregnancy hypertension | 0.89 (0.59, 1.33) | 0.98 (0.64, 1.49) | 0.89 (0.58, 1.37) | 1.01 (0.65, 1.59) | 1.08 (0.69, 1.67) | 1.13 (0.71, 1.79) |
| Gestational hypertension | 1.44 (1.08, 1.92) | 1.45 (1.08, 1.94) | 1.53 (1.14, 2.05) | 1.57 (1.16, 2.11) | 1.52 (1.11, 2.10) | 1.50 (1.08, 2.07) |
| Eclampsia | 1.35 (0.59, 3.09) | 1.24 (0.54, 2.84) | 1.38 (0.58, 3.26) | 1.27 (0.54, 2.99) | 1.76 (0.76, 4.07) | 1.60 (0.69, 3.71) |
| In-hospital delivery | 0.86 (0.51, 1.45) | 0.86 (0.51, 1.45) | 0.94 (0.51, 1.74) | 0.85 (0.45, 1.60) | 0.51 (0.27, 0.95) | 0.57 (0.29, 1.09) |
| Attendant |  |  |  |  |  |  |
| Doctor of Medicine | 1.00 (Reference) | 1.00 (Reference) | 1.00 (Reference) | 1.00 (Reference) | 1.00 (Reference) | 1.00 (Reference) |
| Doctor of Osteopathy | 0.82 (0.57, 1.19 | 0.81 (0.56, 1.17) | 0.82 (0.56, 1.19) | 0.79 (0.54, 1.15) | 0.79 (0.50, 1.22) | 0.75 (0.48, 1.17) |
| Certified Nurse Midwife/Other | 0.84 (0.53, 1.33) | 0.80 (0.50, 1.27) | 0.76 (0.47, 1.22) | 0.73 (0.45, 1.19) | 0.93 (0.55, 1.56) | 0.80 (0.47, 1.36) |
| *Note*: RR = risk ratio. ^a^ Adjusted for all other risk factors in the table. Antepartum stillbirths are the comparison group. ^b^ Includes prior live births, now living or dead. ^c^ Maternal tobacco use at any time during pregnancy. ^d^ Underweight (<18.5), Normal (18.5-24.9), Overweight (25-29.9), Obese class I (30-34.9), Obese class II (35-39.9), Obese class III (≥40). ^e^ Individuals for whom Hispanic ethnicity was unknown were assumed to be non-Hispanic.  To examine the potential mechanisms associated with missingness of timing of fetal death, we ran a bivariate log-binomial regression where missing or unknown foetal death was coded as 1 and valid data coded as 0. We found those missing outcome data were more likely to have missing data on covariates, specifically educational attainment, number of prior live births, timing of first prenatal care visit, smoking during pregnancy, and pre-pregnancy body mass index. | | | | | | |
